# Identification of Antibacterial Activity of *Hepcidin* From Antarctic Notothenioid Fish

**DOI:** 10.3389/fmicb.2022.834477

**Published:** 2022-04-12

**Authors:** Mingli Liu, Ruiqin Hu, Wenhao Li, Wenyi Yang, Qianghua Xu, Liangbiao Chen

**Affiliations:** ^1^Key Laboratory of Exploration and Utilization of Aquatic Genetic Resources, Ministry of Education, Shanghai Ocean University, Shanghai, China; ^2^International Research Center for Marine Biosciences, Ministry of Science and Technology, Shanghai Ocean University, Shanghai, China

**Keywords:** *hepcidin*, Antarctic notothenioid fish, antibacterial peptide, recombinant protein, antibacterial activity

## Abstract

*Hepcidin* is a small peptide composed of signal peptide, propeptide, and the bioactive mature peptide from N terminal to C terminal. Mature *hepcidin* is an antibacterial peptide and iron regulator with eight highly conserved cysteines forming four intramolecular disulfide bonds, giving it a β sheet hairpin-like structure. *Hepcidin* homologs are found in a variety of vertebrates, especially fish, and their diversity may be associated with different habitats and different levels of pathogens. *Dissostichus mawsoni*, an Antarctic notothenioid fish that lives in the coldest water unlike most places of the world, with at least two *hepcidin* variants with eight cysteines. We confirmed the formation process of activated mature *hepcidins* from *D. mawsoni* in Chinese hamster ovary (CHO) cell line, obtained recombinant *hepcidin* protein from prokaryotes, and characterized its binding ability and antibacterial activity against varying bacteria. The expression of *hepcidin* in CHO cell line showed that the prepropeptide of *Dmhep_8cysV1* and *Dmhep_8cysV2* cleavage into smaller mature peptide. The antibacterial assay and flow cytometry showed that *Dmhep_8cysV1*, *Dmhep_8cysV2*, and *Drhep* bound to different bacteria and killed them with different minimum inhibitory concentration. These data suggest that *hepcidin* plays an important role in the innate immunity of *D. mawsoni* and is of great value in improving resistance to pathogens.

## Introduction

Since the 1950s, antibiotics have been widely used around the world to fend off pathogens. Unfortunately, multidrug-resistant microbes have evolved at an unprecedented rate and are insensitive to many current treatment (Pei et al., 2019). In the 21st century, people have increasingly turned to study plants and animals in the hope of identifying and characterizing antimicrobial peptides with broad-spectrum antimicrobial activity and unique membrane-directed mechanism to replace traditional antibiotics (Iman et al., 2019). So far, thousands of antimicrobial peptides have been discovered from different species; however, their use has not been widely promoted because of their high production costs.

*Hepcidins*, a group of cysteine-rich peptides, are widespread in all kinds of animals. It was initially identified as a natural antibacterial peptide from human urine and highly expressed in liver (Krause et al., 2000; Park et al., 2001); subsequently, it was discovered to be an important iron homeostasis regulator in mammals (Fleming and Sly, 2001; Nicolas et al., 2001; Weinstein et al., 2002). *Hepcidin* polypeptide has been demonstrated to regulate iron release from cells by binding to its receptor, an iron transport membrane protein, ferroportin, which results in its degradation (Nemeth et al., 2004; De Domenico et al., 2007; Fleming, 2008). However, the detailed mechanism of the antibacterial function of *hepcidin* still remains to be studied further.

*Hepcidins* have been identified and predicted in many vertebrates, including fishes, amphibians, reptiles, mammals, and birds. All known hepcidin peptides, containing 84–91 amino acids (aa), comprise signal peptide, propeptide, and the bioactive mature peptide from N terminal to C terminal. There are eight conserved cysteine residues in almost all *hepcidin* mature peptides, which form four intramolecular disulfide bonds that stabilize a βsheet hairpin-like structure (Hunter et al., 2002; Lauth et al., 2005; Ganz and Nemeth, 2006). Researchers have demonstrated that the disulfide bonds are necessary for *hepcidin* to kill bacteria (Hocquellet et al., 2012; Lin et al., 2014). There are many reported researches focused on fish *hepcidin*. Cysteine-rich *hepcidins* have been isolated and identified in fish including large yellow croaker (*Pseudosciaena crocea*) and bass (Shike et al., 2002; Zhang et al., 2009). In addition, the antibacterial activities of recombinant or synthesized *hepcidin* from at least 21 fish species have been tested, including large yellow croaker (*P. crocea*) (Wang et al., 2009), Japanese flounder (*Paralichthys olivaceus*) (Srinivasulu et al., 2008), tilapia (*Oreochromis mossambicus*) (Huang et al., 2007), Nile tilapia (*Oreochromis niloticus*) (Abdelkhalek et al., 2020; Yin et al., 2022), gilthead seabream (*Sparus aurata*) (Cuesta et al., 2008), orange-spotted grouper (*Epinephelus coioides*) (Zhou et al., 2011), black porgy (*Acanthopagrus schlegelii*) (Yang et al., 2011), medaka (*Oryzias melastigmus*) (Cai et al., 2012), Chinese rare minnow (*Gobiocypris rarus*) (Ke et al., 2015), convict cichlid (*Amatitlania nigrofasciata*) (Chi et al., 2015), caspian trout (*Salmo caspius*) (Iman et al., 2019), trout (álvarez et al., 2014), zebrafish (*Danio rerio*) (Lin et al., 2014), pallas (*Brachymystax lenok*) (Xu et al., 2018), medium carp (*Puntius sarana*) (Das et al., 2015), brown trout (*Salmo trutta*) (Huang et al., 2019), roughskin sculpin (*Trachidermus fasciatus*) (Liu et al., 2017), simensis crocodile (*Crocodylus siamensis*) (Hao et al., 2012), and hybrid fish (*Carassius auratus*) (Luo et al., 2020), channel catfish (*Ictalurus punctatus*) (Tao et al., 2014), Mudskipper (*Boleophthalmus pectinirostris*) (Chen et al., 2018), and there is evidence that the antibacterial function of *hepcidin* is conserved in fish. Moreover, *hepcidin* sequences have been cloned from various fishes without testing their antibacterial activity, for example, olive flounder (*P. olivaceus*) (Kim et al., 2005), Atlantic cod (*Gadus morhua*) (Solstad et al., 2008), and Antarctic tooth fish (*Dissostichus mawsoni*) (Xu et al., 2008).

Although *hepcidin* gene widely is distributed in vertebrates, and its number varies across species, from one to seven. It has been identified that there is only one *hepcidin* gene in human, two in mice, five in an Antarctic fish *D. mawsoni* (Xu et al., 2008), and seven in black sea bream (Yang et al., 2007). Among them the *D. mawsoni* (NCBI: txid6530, Fishbase ID: 7039) widely is distributed in freezing waters of high-latitude Antarctic coasts, as far south as 77.5°S (McMurdo Sound), the southern limit of Antarctic marine life. Despite that *D. mawsoni* grows slow in the cold habitat, it can still grow to enormous size (2 m in length and 140 kg in weight), which means they have a strong immune system (Chen et al., 2019). Five variants of *hepcidin* were identified in *D. mawsoni* genome by sequencing (NCBI: EU221590.1, EU221595.1, EU221596.1, EU221602.1, and EU221603.1), and their amino acid sequences are shown in Supplementary Figure 1. One of the conserved variants is named *Dmhep_8cysV1* (EU221590), whereas the rest are newly discovered in genome, such as *Dmhep_8cysV2* (EU221595). However, as a common freshwater model organism, there is only one conserved *hepcidin* in the genome of *D. rerio* (NCBI: txid7955, Fishbase ID: 1822), named *Drhe*p (NCBI: NC_007127.7). Studies have shown that zebrafish recombinant *hepcidin* peptide can inhibit the growth of *Escherichia coli*, *Vibrio anguillarum*, *Staphylococcus aureus*, and *Bacillus subtilis* (Lin et al., 2014). However, whether non-conserved *hepcidin* from *D. mawsoni* has antibacterial activity remains unclear. Therefore, our study determined and compared the antibacterial activity of *hepcidins* in *D. mawsoni* and *D. rerio*.

## Materials and Methods

### Fish and Bacterial Strains

All zebrafish and *D. mawsoni* procedures were approved by Shanghai Ocean University Animal Care and Use Committee. The AB zebrafish strains were purchased from Shanghai Institute of Biology, Chinese Academy of Sciences. The light cycle was maintained at 10-h light off and 14-h light on. The *D. mawsoni* was collected from McMurdo Sound. *Aeromonas hydrophila*, *E. coli*, *Edwardsiella tarda*, *S. aureus*, and *Streptococcus agalactiae* are cultured in Luria broth (LB) medium at their optimum growth temperature.

### RNA Isolation and Tissue Expression Analysis

Various tissues of adult fish were dissected according to the method as previously described (Tripti and Mary, 2010). Tissues were rapidly frozen in liquid nitrogen and stored at −80°C until use. Total RNA for cloning and quantitative reverse transcription polymerase chain reaction (qRT-PCR) was extracted from fish brain, liver, gill, ovary, muscle, skin, heart, gut, kidney, and spleen using TRIzol reagent (Invitrogen) following the manufacturer’s instructions. RNA quality was assessed by electrophoresis on a 1% agarose gel, and RNA concentration was estimated using a Nanodrop 2000 spectrophotometer (Thermo Fisher).

One microgram of total RNA was reverse transcribed using PrimerScript™ RT reagent Kit with gDNA Erase (Takara). qRT-PCR was carried out with FastStart SYBR Green Master (Roche), and each reaction was triplicated to avoid possible random variations. Primers were designed according to various *hepcidin* sequences obtained from the National Center for Biotechnology Information (NCBI). Primers are shown in Table 1.

**TABLE 1:** Primers for quantification of *D. mawsoni* and *D. rerio hepcidin* mRNA by real time quantitative PCR.

Primer	Sequence (5′–3′)
qDrhepF	GACTGAAGCTGAACACAGACTAA
qDrhepR	GCAGTATCCGCAGCCTTTAT
qDmhep_8cysV1F	TGTTTTCCCTATGGAGTGCCC
qDmhep_8cysV1R	CTGCCTGATGTGATTTGGCAT
qDmhep_cysV2F	TTACTGAACACGAGGAGCCC
qDmhep_8cysV2R	TTGTGCAGCACGTTTGACAG
qDrβactinF	GATCTGGCATCACACCTTCTAC
qDrβactinR	TCTTCTCTCTGTTGGCTTTGG
qDmβactinF	ATTGTGACCAACTGGGATGA
qDmβactinR	GGGCAACTCTCAGCTCGT

*Dm, D. mawsoni; Dr, D. rerio; hep, hepcidin; cys, cysteine.*

### Sequence and Phylogenetic Analyses

The *hepcidin* coding sequences (CDS) sequences were analyzed using DNAMAN8 software, and the deduced amino acid sequences were translated using online website, https://web.expasy.org/translate/. The theoretical isoelectric point and molecular weight of the protein were calculated using online website, https://web.expasy.org/compute_pi/. The domain prediction of hepcidin amino acid sequence was executed in SMART.^1^ The deduced amino acid sequences of hepcidin in other species were identified in NCBI GenBank.^2^ Multiple sequence alignment was created with DNAMAN8 software.

### Recombinant Plasmid Construction

*Hepcidin* CDS sequences were inserted into a plasmid with zebrafish β*-actin* promoter at *Eco*RI and *Bam*HI sites. EGFP and mCherry DNA sequence was inserted into CDS regions following 5**′** terminal of propeptide and 3**′** terminal of mature peptide by PCR and In-Fusion Cloning, respectively. These plasmids were transfected into Chinese hamster ovary (CHO) cells using transfection reagent (Thermo Fisher). The hepcidin mature sequences were inserted into pHis-TEV using *Nco*I and *Xho*I enzyme digesting sites. The recombinant plasmid with His tag sequence at the 5**′** terminal of mature *hepcidin* sequence can be transformed into *E. coli* to produce *hepcidin* protein controlled by T7 promoter. All the recombinant plasmids were verified by sequencing.

### Expression of *Hepcidin* in Chinese Hamster Ovary Cell Line

Seed CHO cells had 70–90% confluence for transfection. Diluting 6 μL turbofect reagent and 4 μg plasmid in 100 μL Opti-MEM medium (Gibco), respectively. Then, the two diluting solutions were mixed and incubate for 15 min at room temperature. The mixture was added to cells and shaken gently. The cells were imaged under the fluorescence microscopy 72 h after transfection.

### Expression and Identification of *Hepcidin* in *Escherichia coli*

The recombinant pHis-TEV plasmids were transformed into BL21(DE3) and cultured in ampicillin resistance LB plate at 37°C. A single colony containing various recombinant plasmid was transferred into 4 mL ampicillin (20 μg/mL)–resistant LB medium and cultured with 200 revolutions/min (rpm) shaking at 37°C for 12 h. Three milliliters of the cultures was transferred into 300 mL fresh LB medium containing ampicillin and incubated at 37°C until the OD_600_ reached 0.5, and then isopropyl thiogalactopyranoside was added with a final concentration 0.05 and 0 mM, respectively. The cultures were grown at 18°C for 16 h to induce the expression of the recombinant protein better. The cells were collected, centrifuged at 6,000 *g* at 4°C for 10 min, and resuspended in phosphate-buffered saline (PBS) buffer (Hyclone) for 15 min with 100-W ultrasound in an ice water bath for completely crack of the bacteria. The lysate was centrifuged at 12,000 *g* at 4°C for 20 min, and the precipitation was resuspended in 8 M urea (Sigma), and the supernatant was collected by centrifugation at 4°C after 10 min in ice bath with 100-W ultrasound. Then 20 μL of PBS lysate and urea lysate were respectively taken for Western blot detection. The total protein sample was separated by 15% sodium dodecyl sulfate (SDS)–polyacrylamide gel electrophoresis gel and transferred to 0.22-μm polyvinylidene difluoride membranes (Millipore Corporation) and then blocked with TBST buffer (50 mM Tris–HCl, 150 mM NaCl, and 0.1% Tween20, pH 7.4) containing 5% non-fat milk (wt/vol) at 4°C overnight. After washing with TBST three times for 30 min, the membrane was incubated with mouse anti-His Ab (1:1,000) for 2 h at room temperature. The membrane was washed thrice with TBST for 30 min and then incubated with the horseradish peroxidase–conjugated goat anti-mouse immunoglobulin G Ab (1:3,000) for 1 h at room temperature. After washing with TBST, the membrane was visualized with SuperSignal™ West Pico PLUS Chemluminescent Substrate (Thermo) and imaged using Amersham imager 600 (GE).

### Purification of Recombinant *Hepcidin* Protein

The 8 M urea lysate was purified using a protein purification system (AKTA Pure, GE) with Ni-NTA Resin column. Equal volume of 8 M urea containing 0.4% PEG2000, 0.2 mM GSSG, 2 mM GSH, 2% L-arginine, and 10% glycerol was added into the eluted protein. Then, the eluted protein was treated with gradient dialysis in buffer (0.2% PEG2000, 0.1 mM GSSG, 2 mM GSH, 5% glycerol, 1% L-arginine, 0.1 M EDTA, and 6 or 4 or 3 or 2 or 1 or 0 M urea) of different urea concentrations. Finally, various *hepcidin* proteins were concentrated by using the concentration tube under 3,000 rpm at 4°C. The concentrated proteins were detected using Coomassie blue staining.

### Antibacterial Assay

Antibacterial activity experiment was performed using microtiter broth dilution method; 100 μL test bacteria strains were diluted to approximately 3 × 10^6^ colony-forming units/mL, and then 100 μL diluted peptide was added to 96-well microplates, and PBS (pH 7.4) was used as a control. The mix was incubated in a 37°C incubator. The bacterial growth curve was plotted by measuring the absorbance at 600 nm (OD_600_). The OD_600_ values were measured and recorded, which began when bacteria and peptide were mixed, starting with 0 h and then every hour for 12 h. Survival rate was calculated as the cell density in the presence of *hepcidin* peptides compared with the cell density of control. Assay was performed three times, and all data are presented as mean ± SD.

### Flow Cytometry Analysis of Bacterial Immunofluorescence

The 3 × 10^5^ bacteria cells were treated with 22 μM *Dmhep_8cysV1* protein, 19 μM *Dmhep_8cysV2* protein, and 18 μM *Drhep* protein for 1 h, respectively. Then, bacteria cells were collected at 3,000 rpm for 5 min and washed with PBS buffer twice in PBS (pH 7.4). Then, 100 μL 75% ethanol was added to the bacteria, incubated for 30 min, centrifuged, discarded the supernatant, and washed cells with PBS twice. One hundred microliters of 0.1% Triton X-100 was added and incubated for 45 min; the cells were washed three times in PBS. The cells were incubated in PBS containing 100 μg/mL lysozyme and 5 mM EDTA for 30 min; the cells were washed three times in PBS. One hundred microliters 0.5% blocking reagent solution (Sigma) was added and incubated for 30 min; 1 μL mouse anti-His Ab (1:100) was directly added and shaken for 2 h at 37°C. The suspension was centrifuged and discarded; the cells were washed three times in PBST (PBS containing 0.05% Tween-20); 100 μL 0.5% blocking buffer was added containing goat anti-mouse Alexa Fluor 488 ab (1:500) for 30 min at 37°C. The suspension was centrifuged and discarded; the cells were washed three times in PBST and once in PBS; the cells were resuspended in PBS. Finally, the fluorescence signal of cells was analyzed is using Accuri C6 (Becton, Dickinson and Company, BD). The data were analyzed with FlowJo software.

## Results

### Phylogenetic Analysis of Different *Hepcidin* Variants of *Dissostichus mawsoni* and *Danio rerio*

**FIGURE 1:**
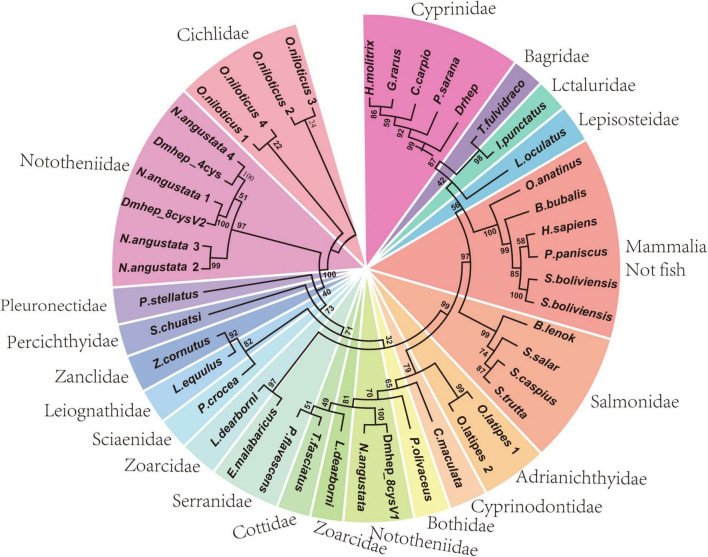
Phylogenetic analysis of *hepcidins* from teleost and other vertebrates. The phylogenetic trees constructed by neighborhood join method in MEGA 7.0. The numbers given are frequency (%) of a given branch in 1,000 bootstrap replications.

To determine the phylogenetic relation of *hepcidins* from *D. mawsoni* and *D. rerio*, a phylogenetic tree was constructed with CDS sequence of *hepcidins* of teleost and other species (Figure 1). The results show *D. rerio* and *Cyprinidae* hepcidin in a clade position, whereas two *hepcidins* from *D. mawsoni* (*Dmhep_8cysV1* and *Dmhep_8cysV2*) were in the same branch position with those of *Notothenia angustata* (Nototheniidae), respectively. The phylogenetic relationship of *hepcidins* between *D. mawsoni* and *D. rerio* was relatively distant.

### Sequence Alignment of Different *Hepcidin* Variants of *Dissostichus mawsoni* and *Danio rerio*

**FIGURE 2:**
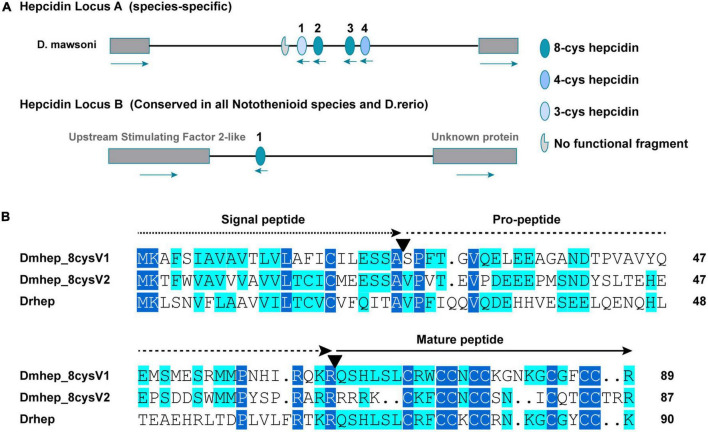
Sequence characteristics of *hepcidin* variants. **(A)** The distribution of conserved and non-conserved *hepcidin* variants in the genome. **(B)** Multiple amino acid alignment and comparison of the different *hepcidin* variants from *D. mawsoni* (*Dmhep_8cysV1* and *Dmhep_8cysV2*) and *D. rerio* (*Drhep*). The three functional regions of the prepropeptide are represented by different lines at the top of each region. The number of amino acids for each variant is shown at the end of each sequence. The putative cleavage sites of the signal peptide and the mature *hepcidin* are marked with inverted triangles. Levels of amino acid conservation are highlighted by dark blue (identical) and light blue (two variations).

Whole genome sequencing analysis revealed that the *hepcidin* gene of *D. mawsoni* is distributed at both genomic loci A and B, and the most different between them is the number of cysteines, ranging from three to eight (Figure 2A). The *hepcidin* genes of *D. mawsoni* named *Dmhep_8cysV1* are located at locus A and *Dmhep_8cysV2* at locus B, respectively, and the only one from *D. rerio* named *Drhep* is also at locus B. There are eight cysteines in all of the three. Their Genbank accession numbers are EU221590.1, EU221595.1, and NC_007127.7. Their CDSs were cloned, sequenced, and aligned. The CDS lengths of *Dmhep_8cysV1*, *Dmhep_8cysV2*, and *Drhep* were 273, 267, and 276 bp, respectively. The full lengths of the three *hepcidin* peptides are 90, 88, and 91 aa, although their signal peptides are 24 aa, the mature region contain 25, 23, and 24 aa, respectively. And the predicted molecular weights of their mature peptide were 2.96, 2.93, and 2.89 KD, and the theoretical isoelectric points were 8.76, 9.37, and 8.94, respectively. Amino acid sequence alignment shows that *Dmhep_8cysV2* is significantly different from the other two, especially from the mature region. However, the mature region of *Dmhep_8cysV1* is highly similar to that of *Drhep*, suggesting that their functions and biological activities may be very similar.

### *Hepcidin* mRNA Expression in *Dissostichus mawsoni* and *Danio rerio*

**FIGURE 3:**
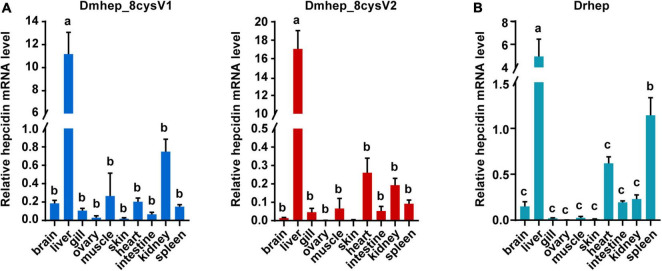
The relative mRNA expression of *hepcidin* variants in different tissues of *D. mawsoni* **(A)** and *D. rerio* **(B)**. Error bars, mean ± SD, *n* = 3 (biological replicates). The same letters (a, b, and c) indicate no significant difference between different tissues, and different letters indicate statistical difference (*p* < 0.05) between different tissues.

To detect the tissue expression level of *hepcidin*, mRNA expression was detected in 10 tissues collected from *D. rerio* and *D. mawsoni*, respectively, including brain, liver, gill, ovary, muscle, skin, heart, gut, kidney, and spleen (Figure 3). Obviously, *hepcidin* mRNA expression was the highest in liver of the two fishes. The mRNA expression level was very low in other tissues, except spleen, which was the secondary tissue with high expression of *Drhe*p, which is responsible for immune and hematopoietic response.

### *Hepcidin* Prepropeptides Were Cleaved Into Mature Peptide and Propeptide *in vitro*

**FIGURE 4:**
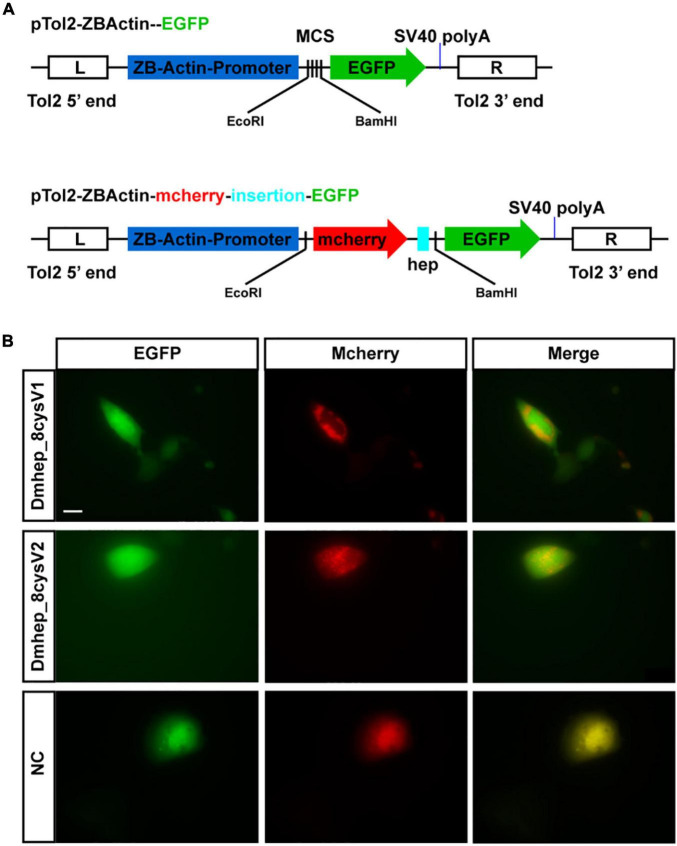
*Hepcidin* prepropeptide were cleaved into mature peptide and propeptide in CHO cell line. **(A)** The structure of recombinant plasmid with or without CDS of *hepcidin* and fluorescent protein. **(B)** The distribution of red and green fluorescent protein in CHO cell line. NC, negative control.

The premature peptide motif “RXK/RR” has been hypothesized to be the cleavage site of furin-like endoproteases, but it has not been demonstrated that the prepeptide and mature peptide can be separated in living cell automatically. To identify that propeptide and mature peptide could be cleaved in live cell, green fluorescent protein tag and red fluorescent protein tag were fused with N-terminal of propeptide and C-terminal of mature peptide, respectively (Figure 4A). The recombinant *hepcidin* plasmid (*Dmhep_8cysV1* and *Dmhep_8cysV2*) was transfected and expressed in CHO cells; microscopic imaging revealed that green fluorescent protein and red fluorescent protein were separated (Figure 4B), whereas the two fluorescent proteins of control plasmid without *hepcidin* sequence were completely fused in CHO cells (Figure 4B). Taken together, the results reveal that *hepcidin* prepropeptides were cleaved into mature peptide and propeptide *in vitro*.

### The Antibacterial Activity of Recombinant Protein of Different *Hepcidin* Variants

**FIGURE 5:**
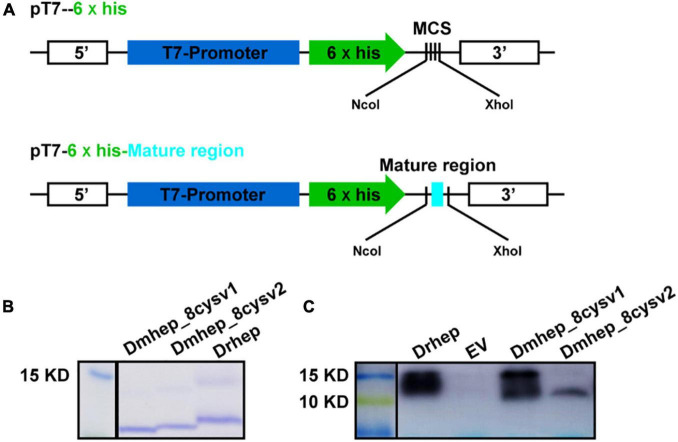
Expression and purification of *hepcidin* in *E. coli*. **(A)** The structure of recombinant plasmid with or without mature region of *hepcidin*. **(B)** Purified *hepcidin* peptide detected by Coomassie blue staining. **(C)** Purified *hepcidin* peptide detected by Western blot.

To figure out whether functional divergence occurred in these sequentially diverse *hepcidin* variants, we examined their antibacterial activity. *Drhep*, *Dmhep_8cysV1*, and *Dmhep_8cysV2* recombinant proteins purified by His tag show a single band about 10 kDa on an SDS polyacrylamide gel by Coomassie blue staining and Western blot (Figure 5).

The purified recombinant protein was constructed in prokaryotic *E. coli* and tested for its antibacterial activities against Gram-positive and Gram-negative bacteria. All three recombinant *hepcidin* peptides showed antibacterial activities. The minimum inhibitory concentration (MIC) of *Drhep* against *E. coli, S. agalactiae*, *A. hydrophila*, and *S. aureus* were 15, 10, 20, and 25 μM, respectively. The MICs of *Dmhep_8cysV1* against *E. coli*, *S. agalactiae*, *A. hydrophila*, and *S. aureus* were 25, 20, 25, and 25 μM, respectively. The MICs of *Dmhep_8cysV2* against *E. coli*, *S. agalactiae*, *A. hydrophila*, and *S. aureus* were 20, 10, 20, and 15 μM, respectively (Table 2). The three *hepcidins* from *D. mawsoni* and *D. rerio* had different degrees of antibacterial activity overall.

**TABLE 2:** Minimum inhibitory concentration of various *hepcidin* peptides.

Bacteria	MIC (μM)		
	*Dmhep_8cysV1*	*Dmhep_8cysV2*	*Drhep*
*S. agalactiae*	20	10	10
*S. aureus*	25	15	25
*E. coli*	25	20	15
*A. hydrophila*	25	20	20

### *Hepcidin* Protein Can Bind to Various Bacteria

**FIGURE 6:**
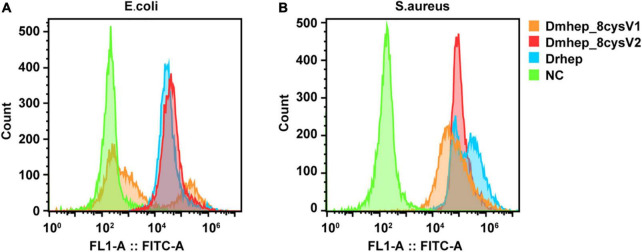
Various *hepcidin* peptides were detected by flow cytometry to bind to *E. coli* **(A)** and *S. aureus* **(B)**. Orange represents *Dmhep_8cysV1* (20 μM), red represents *Dmhep_8cysV2* (20 μM), blue represents *Drhep* (20 μM), and green represents NC (negative control, PBS).

Flow cytometry analysis of bacterial immunofluorescence confirmed *hepcidin* peptide kills bacteria by binding to them. The result showed that all three *hepcidin* proteins can bind to the bacteria at a concentration of 20 μM (Figure 6). The binding capacity of *Dmhep_8cysV2* to *E. coli* was stronger than that of *Dmhep_8cysV1* (Figure 6A), and the binding capacity of *Dmhep_8cysV2* was the same as that of *Drhep*. *Dmhep_8cysV2* also had better binding ability to *S. aureus* than the other two (Figure 6B).

## Discussion

Antimicrobial peptides are one of the important components of fish living in microbe-rich environment (Samuthirapandian et al., 2010). The AMP (antimicrobe peptide) *hepcidin* widely exists in fish and plays an important role in innate immune system of fish (Shi and Camus, 2006). In recent years, a large number of studies have been carried out on the antibacterial activity of AMP, especially on *hepcidin* of various vertebrates, looking forward to find a substitute for antibiotic. The sequences and structure domains of *hepcidin* variants from *D. mawsoni* were analyzed; their recombinant peptides were obtained, and their antimicrobial activities were tested in this study.

The encoded prepropeptide contains 84 aa in human, but only 20- to 25-aa peptides were initially found in urine (Park et al., 2001), meaning that the prepropeptide was cleaved to form a mature peptide. The conserved arginine/lysine propeptide cleavage recognition site (RXRR/RXKR) of furin-like endoproteases was found in all three *hepcidins* (Figure 2B). *Dmhep_8cysV1* and *Dmhep_8cysV2* propeptides separate into two parts in CHO cells (Figure 4B), probably because arginine residues at their carboxyl terminal are cleaved by widespread furin, similar to viral glycoproteins (Garten et al., 1994). This procedure is common with posttranslational modification of membrane and secretory proteins to form a functional peptide. In addition, it is assumed that the full *hepcidin* sequence supplies a codon long enough to ensure that the translation process is performed smoothly and correctly.

Sequence alignment showed that the mature region of *Dmhep_8cysV1* is highly parallel to *Drhep*, but *Dmhep_8cysV2* had low similarities with the other two mature regions except for the eight cysteines in this region. It is believed that the mature peptide of teleost fish is highly conserved (Ke et al., 2015), and the newly evolved *Dmhep_8cysV2* in the genome enables *D. mawsoni* to acquire *hepcidin* sequence diversity. Therefore, whether the diverse sequences of *hepcidin* are helpful to increase the immunity ability for *D. mawsoni* is still unclear. The result showed that mature *hepcidin* could inhibit all kinds of bacteria. In this study, the recombinant peptide of *D. mawsoni* and *hepcidin* of *D. rerio* both inhibited the growth of Gram-positive and Gram-negative bacteria. However, *Dmhep_8cysV2* had a lower MIC against *S. aureus* than both the other two. This may relate to the origin of *Dmhep_8cysV2*, as it is a newly evolved site located at genomic locus A, rather than the ancient locus B such as *Dmhep_8cysV1* and *Drhep*. In addition, the amino acid sequence showed that *Dmhep_8cysV2* was significantly different from *Dmhep_8cysV1* and *Drhep*, which is attributed to the differences in antibacterial ability, for which the latter two show more similarities than the former. Different *hepcidin* variants have different MIC values for some bacteria, but these prove that *hepcidin* has a broad-spectrum antibacterial ability.

Given the special habitat of *D. mawsoni* and its various *hepcidin* variants, the additional function of *hepcidins* deserves further study and exploration. Many studies have shown that *hepcidin* mature peptides form a characteristic antiparallel β-sheet conformation with eight, six, or four conserved cysteines (De Domenico et al., 2008). At the same time, there was evidence that proved *hepcidin* lost antibacterial function if all the eight cysteines were replaced by alanine (Lin et al., 2014). In fact, there are three *hepcidins* in *D. mawsoni*, except for the two mentioned in this article, with the third one containing only four cysteines. Therefore, the relationship between the structure and function of different *hepcidins* variants remains to be explored.

In addition, although many *hepcidins* have a certain degree of antibacterial activity, how they kill bacteria is still not clear. Previous studies have demonstrated that peptides with β sheet structure bind to bacterial membrane to form complexes that kill microorganisms (Huang, 2000; Chen et al., 2003). Flow cytometry showed that *hepcidin* from *D. mawsoni* and *D. rerio* could bind to bacteria and may be the complexes formed in this process. However, structure of the complexes is still unclear, and whether there are other molecules in bacterial cells that can target *hepcidin* needs further study.

## Data Availability Statement

The original contributions presented in the study are included in the article/Supplementary Material, further inquiries can be directed to the corresponding author.

## Ethics Statement

The animal study was reviewed and approved by the Shanghai Ocean University Animal Care and Use Committee.

## Author Contributions

LC managed the project. ML designed and performed experiments and wrote the manuscript. RH contributed to interpretation of data and edits to the manuscript. WY contributed to RNA and strain preparations. WL and QX performed fish and tissue collections and sample preparations. All authors contributed to the article and approved the submitted version.

## Conflict of Interest

The authors declare that the research was conducted in the absence of any commercial or financial relationships that could be construed as a potential conflict of interest.

## Publisher’s Note

All claims expressed in this article are solely those of the authors and do not necessarily represent those of their affiliated organizations, or those of the publisher, the editors and the reviewers. Any product that may be evaluated in this article, or claim that may be made by its manufacturer, is not guaranteed or endorsed by the publisher.

## Footnotes

1
https://smart.embl-heidelberg.de

2
http://www.ncbi.nlm.nih.gov/nucleotide/
