# Supplementary figures and images for: Identification of Antibacterial Activity of Hepcidin From Antarctic Notothenioid Fish
Source: Front Microbiol. 2022 Apr 12;13:834477. doi: 10.3389/fmicb.2022.834477 (PMC9039748; doi:10.3389/fmicb.2022.834477)

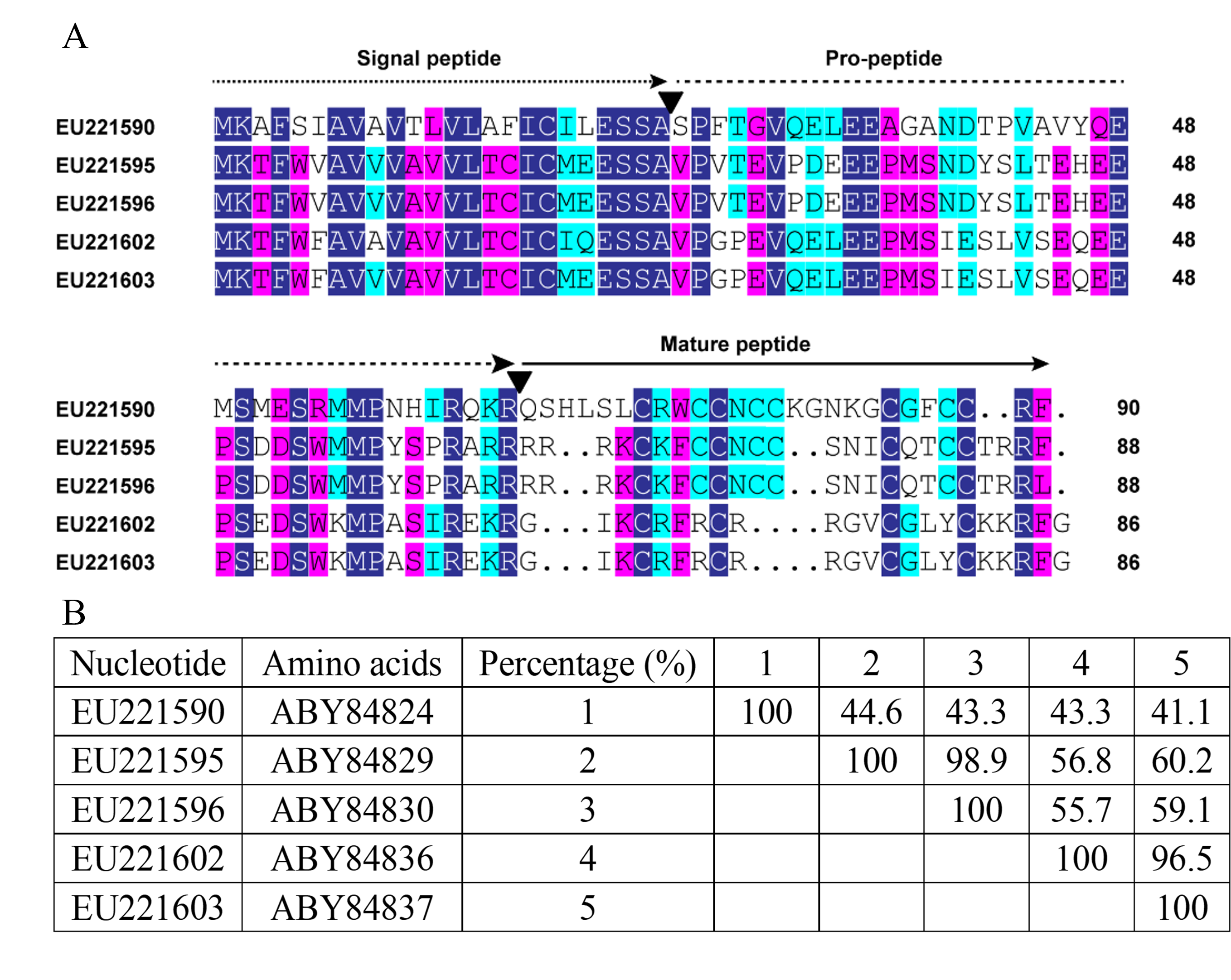

Supplement: Supplementary file 1 [file Image_1.tif]

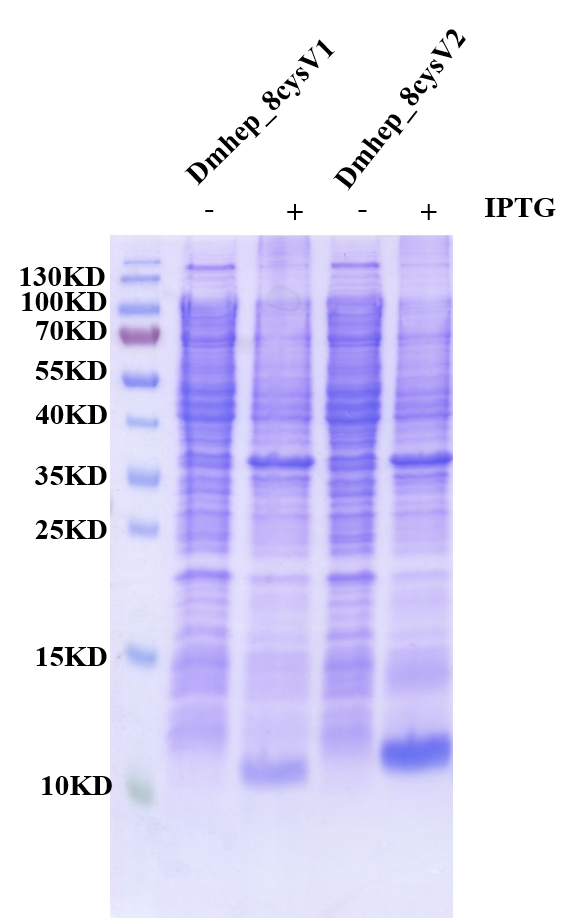

Supplement: Supplementary file 2 [file Image_2.tif]
